# Impact of Perioperative Antibiotic Prophylaxis Targeting Multidrug-Resistant Gram-Negative Bacteria on Postoperative Infection Rates in Liver Transplant Recipients

**DOI:** 10.3390/diagnostics15151866

**Published:** 2025-07-25

**Authors:** Eleni Massa, Dimitrios Agapakis, Kalliopi Tsakiri, Nikolaos Antoniadis, Elena Angeloudi, Georgios Katsanos, Vasiliki Dourliou, Antigoni Champla, Christina Mouratidou, Dafni Stamou, Ioannis Alevroudis, Ariadni Fouza, Konstantina-Eleni Karakasi, Serafeim-Chrysovalantis Kotoulas, Georgios Tsoulfas, Eleni Mouloudi

**Affiliations:** 1 Intensive Care Unit, General Hospital “Hippokratio”, 54642 Thessaloniki, Greece; elenizioga@yahoo.com (E.M.); kalliopi.tsakiri@gmail.com (K.T.); vicky_dourliou@hotmail.com (V.D.); antigoni_h@windowslive.com (A.C.); chris1mourat@gmail.com (C.M.); stdafni@hotmail.com (D.S.); giannis.alevroudis@gmail.com (I.A.); akiskotoulas@hotmail.com (S.-C.K.); elmoulou@yahoo.gr (E.M.); 2 Department of Internal Medicine, General Hospital “Agios Pavlos”, 55134 Thessaloniki, Greece; dimagap@yahoo.gr; 3 Transplantation Surgery Clinic, “Hippokratio” University Hospital, 54642 Thessaloniki, Greece; nikanton@auth.gr (N.A.); georgios.katsanos@gmail.com (G.K.); ariadnefou@gmail.com (A.F.); ke_karakasi@hotmail.com (K.-E.K.); tsoulfasg@gmail.com (G.T.)

**Keywords:** perioperative antibiotic prophylaxis, multidrug-resistant Gram-negative bacteria, outcome, liver transplantation

## Abstract

Infections with multidrug-resistant (MDR) organisms remain a significant cause of morbidity and mortality among liver transplant recipients, despite advances in surgical techniques and immunosuppressive therapy. This prospective observational study aimed to evaluate the impact of targeted perioperative antibiotic prophylaxis against MDR Gram-negative bacteria on postoperative infections and mortality in liver transplant recipients. Seventy-nine adult patients who underwent liver transplantation and were admitted to the ICU for more than 24 h postoperatively were included. Demographics, disease severity scores, comorbidities, and lengths of ICU and hospital stay were recorded. Colonization with carbapenem-resistant Gram-negative bacteria was assessed via preoperative and postoperative cultures from the blood, urine, rectum, and tracheal secretions. Patients were divided into two groups: those with MDR colonization or infection who received targeted prophylaxis and controls who received standard prophylaxis. Infectious complications (30.4%) occurred significantly less frequently than non-infectious ones (62.0%, *p* = 0.005). The most common infections were bacteremia (22.7%), pneumonia (17.7%), and surgical site infections (2.5%), with most events occurring within 15 days post-transplant. MDR pathogens isolated included *Klebsiella pneumoniae*, *Acinetobacter baumannii*, and *Pseudomonas aeruginosa*. Although overall complication and mortality rates at 30 days and 3 months did not differ significantly between groups, the targeted prophylaxis group had fewer infectious complications (22.8% vs. 68.5%, *p* = 0.008), particularly bacteremia (*p* = 0.007). Infection-related mortality was also significantly reduced in this group (*p* = 0.039). These findings suggest that identification of MDR colonization and administration of targeted perioperative antibiotics may reduce septic complications in liver transplant patients. Further prospective studies are warranted to confirm benefits on outcomes and resource utilization.

## 1. Introduction

Liver transplantation is one of the most complex surgical procedures, being performed in critically ill patients with end-stage liver disease. Although both quality of life and life expectancy in liver transplant recipients have substantially improved in recent years, complications such as graft rejection and infections remain major contributors to morbidity and mortality in this population.

In recent years, there has been a progressive increase in the number of infections caused by multidrug-resistant (MDR) organisms, defined as bacteria resistant to at least three classes of antibiotics, and extensively drug-resistant (XDR) organisms, with sensitivity to only two or three antibiotics, such as colimycin, tigecycline, or aminoglycosides [1]. These infections represent a major concern worldwide, as they are associated with higher mortality, prolonged hospitalization, and increased healthcare costs [2,3].

Clinically relevant MDR organisms in transplant patients include *Pseudomonas* species, *Acinetobacter baumannii*, and members of the *Enterobacteriaceae* family. The prevalence of MDR organisms among transplant recipients varies by region. Studies from high-prevalence areas report resistance rates ranging from 18% to 50% [4]. Infections caused by MDR organisms are associated with increased morbidity and mortality, as well as a higher risk of graft rejection [5].

Surgical site infections (SSIs) are among the most common complications following surgical procedures. SSIs account for 14–16% of nosocomial infections, with approximately two-thirds involving the incision site and the remainder affecting the underlying tissues and organs accessed during surgery. These latter SSIs are responsible for approximately 77% of mortality among surgical patients [6]. Transplant recipients are more susceptible to SSIs than other surgical patients undergoing similar operations [3,7]. In addition, the incidence of SSIs varies significantly depending on the transplanted organ, ranging from 3 to 53%, with the highest rates observed in small intestine transplants, followed by liver (10–37%), pancreas (10–47%), kidney (4–11%), and heart or lung transplants (4–11%) [8,9].

Furthermore, patients who develop SSIs are up to 60% more likely to require admission to the intensive care unit (ICU), have a fivefold increased risk of hospital readmission, and even minor incisional infections can prolong hospitalization by approximately 10 days [1]. SSIs are associated with significant morbidity and substantially increase healthcare costs. They account for USD 3.2 billion per year in emergency admissions, with an average hospital stay of 11 days per patient. Moreover, SSIs are the leading cause of non-scheduled postoperative readmissions [10,11].

The occurrence of SSIs depends on the site of the surgical incision, the virulence of the microorganisms involved, and the host’s immune response. The discovery of penicillin by Alexander Fleming in 1928 and the subsequent implementation of antimicrobial prophylaxis in surgical patients marked a major milestone in the prevention of SSIs during the 20th century [12]. However, the widespread and inappropriate use of antibiotics has contributed to antimicrobial resistance, which remains a major clinical challenge. Despite the broad use of prophylactic antimicrobials, severe SSIs persist and have not been fully eliminated.

Identifying patients colonized with MDR organisms preoperatively can help guide the adaptation of measures to prevent the spread of infection after liver transplantation and optimize the prescription of immunosuppressive therapy [13,14]. Several studies have highlighted the importance of assessing microbial colonization in liver transplant candidates, emphasizing its value in anticipating and managing post-transplant infections. Data from surveillance cultures can assist in both the prediction and treatment of infections in these patients [15].

However, no studies to date have specifically evaluated the impact of targeted perioperative chemoprophylaxis in patients colonized with MDR organisms undergoing liver transplantation. When a liver transplant recipient is colonized or receiving treatment for an active infection at the time of transplantation, the chemoprophylaxis regimen should be reevaluated, as it is known that colonization with certain microorganisms increases the risk for SSIs. In a study published in 2018 [16], colonization with any organism was an independent risk factor for infection in liver transplant patients.

It remains unclear whether treating microbial colonization provides a significant clinical benefit, and until recently, there were no formal recommendations for the routine screening of all liver transplant candidates or recipients for MDR organisms. Although the most recent ESCMID/EUCIC guidelines on perioperative antibiotic prophylaxis in patients colonized with MDR organisms recommend screening liver transplant candidates for colonization as good clinical practice, there is insufficient evidence to support or refute the use of targeted chemoprophylaxis in this context [17].

The aim of the present study is to document colonization and latent infections in both liver transplant donors and recipients and to evaluate whether targeted perioperative chemoprophylaxis based on colonization status and culture results can prevent post-transplant infections caused by carbapenem-resistant MDR organisms.

## 2. Materials and Methods

This was a prospective observational study. No randomization was applied. Group assignment was based solely on colonization status as determined by preoperative microbial cultures.

The study was conducted in the ICU of the General Hospital of Thessaloniki “Ippokrateio” in collaboration with the Transplant Clinic of the Aristotle University of Thessaloniki (AUTH) from 1 January 2017 to 31 August 2020. A total of 79 adult patients who underwent liver transplantation and remained in the ICU for more than 24 h postoperatively were included.

Demographic data (age, sex, height, and weight) were recorded for all participants (donors and recipients), along with medical history, comorbidities, length of hospital and ICU stay, and the underlying cause of liver cirrhosis. In addition, the APACHE II (Acute Physiology and Chronic Health Evaluation II), SOFA (Sequential Organ Failure Assessment), and MELD (Model for End Stage Liver Disease) scores were calculated to assess disease severity in the transplant recipients.

Baseline cultures of blood, urine, rectal swabs, and bronchial secretions were obtained from all participants to detect colonization by Gram-negative carbapenem-resistant organisms. Samples were collected during the preoperative evaluation at the Transplant Clinic, upon ICU admission postoperatively, and on hospital days 7, 14, 21, and 28, as well as whenever infection was suspected. Additionally, routine laboratory blood tests and inflammatory markers (C-reactive protein [CRP] and procalcitonin [PCT]) were measured daily during the first three postoperative days and subsequently on the same days as culture collection.

Cultures were processed according to standard microbiological protocols. Rectal swabs were screened using CHROMagar KPC for the detection of CRE. Bronchial specimens were plated on MacConkey and blood agar for the detection of Gram-negative pathogens, including *Klebsiella pneumoniae*, *Acinetobacter baumannii*, and *Pseudomonas aeruginosa*.

### 2.1. Comparison of Study Groups

Colonization was defined as the detection of MDR Gram-negative bacteria in surveillance cultures (rectal swab, bronchial secretions, etc.) without signs of clinical infection. Latent infection referred to the presence of a previously treated infection or microbial presence with minimal inflammatory response.

The liver transplant recipients were divided into two groups. Group A was the study group and included patients who were colonized with MDR Gram-negative organisms, had latent infections, or received an organ from a donor colonized with Gram-negative microbes, as determined by the culture results and the minimum inhibitory concentration (MIC) from antibiotic susceptibility testing. Group B (control group) comprised the remaining participants. Patients in Group A received targeted perioperative prophylaxis (meropenem 2 g + colimycin 10 million units ± tigecycline 200 mg), while patients in Group B received standard antibiotic prophylaxis. The frequency, type, and timing of postoperative infections were recorded and compared between the two groups.

Specifically, meropenem was administered intravenously at 2 g every 8 h, colimycin at a 10 million IU loading dose followed by 5 million IU every 12 h, and tigecycline with a 200 mg loading dose followed by 100 mg daily, all intravenously. Antibiotic administration began intraoperatively and continued for 1–2 days postoperatively. Treatment continued for more days in cases where infection was documented in the recipient.

### 2.2. Statistical Analysis

Statistical analysis of the data was performed using SPSS (Statistical Package for Social Sciences SPSS version 27.0, Chicago, IL, USA). Binary variables between patient groups were evaluated using the χ^2^ test or Fisher’s exact test. For the comparison of quantitative variables, the t-test was used for normally distributed data, and the Mann–Whitney U test for non-normally distributed data. Univariate analysis data were entered into a multivariate forward stepwise logistic regression model and analysis was performed to calculate the odds ratio (OR) and the 95% confidence intervals (CI). Statistical significance was defined as a value of *p* ≤ 0.05. Survival analysis was performed using the Kaplan–Meier curve.

## 3. Results

A total of 79 patients were eligible for this study, of whom 61 were males and 18 were females. The mean age of the study population was 53 years for recipients (25 to 70) and 55 years (17 to 69) for the donors. The most common cause of cirrhosis was alcoholic liver disease, hepatocellular carcinoma, hepatitis B and C, and nonalcoholic steatohepatitis (NASH).

Among the donors, 59 were male and 20 were female. The reported causes of death included traumatic brain injury, subarachnoid hemorrhage, and cardiac arrest.

The mean ICU length of stay immediately following transplantation was 4 days (range: 1–30). The calculated disease severity scores were as follows: APACHE II, mean 11 (range: 4–24); admission SOFA, mean 10 (range: 5–18); and MELD score, mean 16 (range: 6–35).

Among the 79 patients (Table 1), 30.4% developed infectious complications and 62% experienced non-infectious complications during their postoperative course, a difference that was statistically significant (*p* = 0.005).

The non-infectious complications included liver dysfunction and graft rejection, arrhythmias, acute coronary syndromes, neurological events including strokes and seizures, postoperative bleeding, portal vein thrombosis or hepatic artery thrombosis, surgical reoperations, acute renal failure, and hemodialysis.

Bleeding was the most common surgical complication (15.1%), and all patients underwent reoperation for hemostasis. Some patients required reoperation for other surgical reasons, such as managing biliary enteric anastomosis complications or retransplantation due to transplant failure.

### 3.1. Infections and Bacterial Profile Among Patients of the Study

Infections were diagnosed based on the CDC/NHSN criteria. Bacteremia required positive blood cultures with systemic signs (fever, chills). Pneumonia was defined by new radiographic infiltrates plus clinical and laboratory findings (fever, purulent sputum, elevated CRP/PCT).

Preoperative screening of the donors and the recipients revealed that 13.9% of the recipients were colonized with *Klebsiella pneumoniae*, *Acinetobacter baumannii*, and *Pseudomonas aeruginosa*, while one was positive for pan-resistant *Acinetobacter baumannii*. Donors were colonized or had recovered from MDR infections after appropriate treatment at a rate of up to 30.4%.

MDR Gram-negative organisms were detected in 35 patients (44.3%), all of whom were assigned to Group A. The remaining 44 patients (55.7%) comprised Group B.

Overall, infectious complications were significantly less frequent than non-infectious complications (*p* = 0.005). Among infectious events, bloodstream infections were the most common (22.7%), followed by pneumonia (17.7%) and surgical site infections (SSIs) (2.5%). The majority of septic events occurred within the first 15 days postoperatively. The predominant Gram-negative pathogens isolated were *Klebsiella pneumoniae* and *Acinetobacter baumannii* (Table 2).

Univariate analysis of infection occurrence on the 7th day post-transplantation identified white blood cell (WBC) count as being marginally associated with infection (*p* = 0.069; OR 1.00, 95% CI 0.95–1.22). Other evaluated parameters—including age, sex, MELD score, APACHE II score, cause of cirrhosis, and serum PCT levels—were not significantly related to infection (Table 3).

The infections that were recorded in both groups on the 7th, 14th, 21st, and 28th day post-transplantation are presented in Table 4. There were no statistically significant differences at any time point between the two groups.

In total, 16 patients (20.25%) developed an infection within the first month after liver transplantation, with some experiencing multiple septic episodes. Overall, 24 sepsis events (30.37%) were recorded, the majority of which occurred within the first 7 days of hospitalization.

Furthermore, most colonizations with MDR organisms postoperatively occurred after the 8th to the 9th day of ICU admission, and in up to 30% of cases, the organisms isolated matched those carried by the donor.

### 3.2. Patient Outcomes

Overall, patient outcomes were favorable. At both 6 and 12 months following liver transplantation, 73.41% of patients remained alive. A total of 21 patients (26.58%) died within the first 6 months: 6 patients (7.59%) from infectious causes—5 within the first month and 1 in the third month—and 15 patients (18.98%) from surgical or immunological causes, including anastomotic leak, bleeding, acute portal vein or hepatic artery thrombosis, graft rejection, liver dysfunction, and graft-versus-host disease. While mortality from infectious versus non-infectious causes differed substantially, this difference did not reach statistical significance (*p* = 0.078). Specifically, infection-related mortality during the first three months was 7.59%, with no significant difference observed between the two groups (Table 5).

### 3.3. Survival Curves

**Figure 1:**
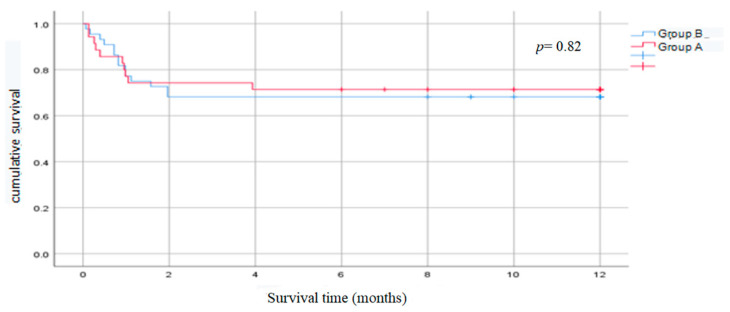
Kaplan–Meier survival curve.

The Kaplan–Meier survival curves show better survival rates for the patients in Group B compared to those in Group A for the first month after transplantation, but then the trend shifts in favor of the patients in Group A just before the second month. This could be due to the low number of deaths in both groups. The survival rate was good in both groups and did not at any point drop below 0.5 (Figure 1).

## 4. Discussion

The present study investigates the impact of colonization and latent infection with MDR Gram-negative bacteria, as well as the role of targeted perioperative antibiotic prophylaxis, in patients undergoing liver transplantation. This topic is particularly relevant in Greece, where MDR organisms are endemic in hospitals and transplant recipients are at increased risk for infection.

Although there is extensive literature regarding SSIs for solid organ transplant patients, there is a lack of clinical trials studying the occurrence and the type of infections in recipients who were colonized with MDRs or were treated for infection caused by MDRs or received a graft from a colonized donor.

The findings of this study aim to enhance our understanding of the safe utilization of organs from donors colonized with MDR organisms, the risk of early post-transplant infections, and the potential benefits of targeted perioperative prophylaxis. The research was conducted at one of the two liver transplantation centers in Greece.

Additionally, transmission of infection from a donor to a recipient is a rare event that is associated with increased morbidity and mortality. It is important to minimize these infections while increasing the chances for donation and improving the safety and outcomes of transplantation [18,19].

The incidence of infections caused by MDR bacteria in transplant patients is rising rapidly worldwide, reaching up to 50% in some centers [20]. Infections due to MDR organisms are associated with lower survival rates compared to infections caused by non-resistant organisms, with mortality reported to be as high as 71% in certain cases. In a Swiss study [21] involving 577 liver transplant recipients, 55% developed an infection within 12 months post-transplantation. Bacteria, viruses, and fungi accounted for 59%, 33%, and 8% of infections, respectively, and 42% of patients died from infection during the study period. Another review of 317 liver transplant recipients [22] found that 45% developed an infection within the first six months post-transplantation, and those with multiple septic episodes had significantly higher mortality.

An Italian study [23] reported that 10.5% of organ donors had latent infections with carbapenem-resistant Gram-negative bacteria, which were only identified after donation. Transmission to recipients occurred in up to 13% of cases, with the highest risk observed when donors had bloodstream infections and the transplanted organ was colonized or infected. In these cases, recipients were often treated with antibiotics that were either inappropriate, initiated late (after donor infections were identified), or administered for an insufficient duration.

In our study, 30.4% of the donors and 13.9% of the recipients had infection or colonization by MDR organisms. Infectious complications from MDR bacteria occurred in 16 transplant patients (20.25%) within the first month after the operation. There were patients who experienced multiple septic episodes. The total number of sepsis events was 24 (30.37%). Recipients in Group A that were prescribed targeted antibiotic prophylaxis experienced sepsis events up to the 7th day following liver transplantation at a rate of only 5.7%. One transplant patient presented with bacteremia from XDR *Klebsiella pneumoniae* and the second one presented with pneumonia with bacteremia from *Acinetobacter baumannii*. In both cases, the grafts were harvested from donors whose microbial isolates differed from those causing infections in the recipients. Specifically, one donor had a bloodstream infection caused by Corynebacterium species, while the other had a urinary tract infection caused by *Enterobacter aerogenes*.

On the other hand, 18.2% of the patients in Group B, who did not receive tailored treatment but received the perioperative prophylaxis recommended by guidelines, presented with infections up to the 7th day. In this group, there were patients who should have received perioperative targeted antibiotics as the graft was from a colonized donor or the donor had a latent infection. In particular, the information about the profile of the microbes of the donor and recipient was available the second and third day after the operation because of misinformation or delayed culture results. As a result, these patients did not receive customized chemoprophylaxis, and this could explain the higher rate of infections in this group. It is noteworthy that out of the eight patients that had an infection, six (75%) presented with an infection involving the same microbes present in the donor or recipient before the transplantation. This observation is very important because it highlights the risk of transmission of MDR organisms from donors to recipients when there is no preoperative screening for colonization or infection. Equally significant is the fact that, although the majority of donors tested negative for bacteremia or infection of the harvested organ, the recipients presented with an infection with the same bacteria within the first 10 days following the transplantation.

In a study by Sanclemente et al. [24] that involved kidney transplantation, it was shown that perioperative tailored prophylaxis reduced the postoperative infections from *Eenterobacteriaceae*, and most importantly, the incidence of extended-spectrum beta-lactamase (ESBL) bacterial strains decreased to one third.

In our study, patients in Group A experienced fewer sepsis events—6 patients (17.1%) with 8 events (22.8%)—compared to Group B, in which 10 patients (22.7%) experienced 16 sepsis events (36.4%); however, the difference was not statistically significant (*p* = 0.195). Bloodstream infections were the most common (22.8%), followed by pneumonia (17.7%), SSIs, specifically bile peritonitis (2.5%), and urinary tract infections (1.3%). Bacteremia was significantly more frequent in Group B (34.1%) compared to Group A (8.6%) (*p* = 0.007). This may be attributed to the fact that patients in Group B received inappropriate intraoperative antibiotics due to the delayed identification of donor and recipient microbial profiles. The Gram-negative bacteria isolated included *Klebsiella pneumoniae* (20.3%), *Acinetobacter baumannii* (19%), *Pseudomonas aeruginosa* (6.3%), *Stenotrophomonas maltophilia* (5.1%), and *Chromobacterium sakazakii* (1.3%).

In our study, *Klebsiella pneumoniae* was the most frequently identified MDR organism, followed by *Acinetobacter baumannii*, which is consistent with findings reported in the current literature [15,16,17,18,19,20,21,22,23,24,25,26]. Additionally, most patients experienced their first sepsis episode within the first 7 days post-transplantation, and these cases were predominantly among recipients of grafts from infected or colonized donors. Similar observations have been made by Giannella et al. [26], who reported that carbapenem-resistant *Enterobacteriaceae* (CRE) infections typically occur early—within the first month post-transplantation—and are healthcare-associated. Very early onset of infection, within 7–14 days, has been particularly noted in recipients of grafts from donors colonized with CRE at the time of transplantation [27].

With respect to patients’ colonization after the operation and while being in the ICU, it was shown that the patients were colonized at a rate of up to 16.4%. In half of the cases, isolates matched the bacteria found in the donor, and the timing of the positive results was around the 8th to the 9th day in the ICU. The mean time for colonization in the literature is the 10th day [15,16,17,18,19,20,21,22,23,24,25,26,27,28], although the frequency of colonization and infection with CRE could vary depending on the type of transplantation, the local epidemiology, and the policies to reduce their spread.

SSIs after liver transplantation are caused by MDR organisms. In a literature review by Freire M. et al. [29], SSIs were present in 14.5% of transplant patients after their operation, and the most common pathogen was *Klebsiella pneumoniae*. Administering perioperative antibiotics prophylaxis was the sole measure to protect against MDR SSIs. The contribution of antibiotic prophylaxis in reducing SSIs after liver transplantation has been described [30,31]. Frenette et al. [30] note that a higher level of compliance to a modified protocol of antibiotics prophylaxis (vancomycin and ceftriaxone) could reduce the SSIs more than 50%. Furthermore, Ascensio et al. [32] showed that prophylaxis with broad-spectrum antibiotics (glycopeptides and aztreonam) was correlated with lower rates of SSIs compared to monotherapy with cephazoline. Previous studies have primarily focused on broadening the antibiotic spectrum to target Gram-negative bacteria. In one study involving 749 liver transplant recipients [33], 100 of whom were colonized with ESBL-producing *Enterobacteriaceae*, it was observed that the overall colonization rate with ESBL-producing strains was lower among patients who received perioperative prophylaxis with ertapenem.

## 5. Conclusions

In conclusion, screening for MDR organism colonization and administering appropriate antibiotics in liver transplants could lower the number sepsis events in this susceptible group of patients. Although colonization does not equate to active infection, it may represent a significant risk factor for subsequent invasive disease in immunocompromised or critically ill patients, such as liver transplant recipients. In our study, targeted short-course prophylaxis was administered not to treat colonization per se, but to preemptively mitigate the high risk of progression to infection in colonized individuals. Nonetheless, we acknowledge that this approach must be carefully weighed against the principles of antimicrobial stewardship. Further studies are required to validate the use of this practice in improving outcomes, reducing healthcare costs and controlling microbial flora. The choice of the most appropriate antibiotics, as well as the duration of administration, has an impact on infection resolution, cost reduction, and the prevention of resistant bacteria. Antibiotics are a valuable resource that should be used cautiously. Thoughtful use of antibiotics is the best strategy to fight bacterial resistance.

## Figures and Tables

**Table 1:** Overall complications.

	Group A (35)	Group Β (44)	
Non-infectious complications	24/35	25/44	*p* = 0.285 (Chi-squared, χ^2^ = 1.143)
Infectious complications	8/35	16/44	*p* = 0.195 (Chi-squared, χ^2^ = 1.681)
Overall mortality at 30 days	7/35	10/44	*p* = 0.770 (Chi-squared, χ^2^ = 0.086)
Overall mortality at 3 months	0/35	4/44	*p* = 0.125 (Fisher’s exact test)

**Table 2:** Type of infection and pathogens.

Type of Infection	Total (*n* = 79)	Group A (*n* = 35)	Group Β (*n* = 44)	
Pneumonia	14	5	9	*p* = 0.476 (Chi-squared, χ^2^ = 0.509)
Blood stream infections	18	3	15	*p* = 0.007 (Chi-squared, χ^2^ = 7.216)
Surgical site infection	2	1	1	*p* = 0.870 (Fisher’s exact)
Urinary tract infection	1	1	0	*p* = 0443 (Fisher’s exact)
Colonization	13	7	6	*p* = 0.546 (Chi-squared, χ^2^ = 0.574)
Klebsiella pneumoniae	16	5	11	*p* = 0.273 (Chi-squared, χ^2^ = 1.386)
Acinetobacter baumannii	15	5	10	*p* = 0.903 (Chi-squared, χ^2^ = 0.398)
Pseudomonas aeruginusa	5	2	3	*p* = 1.000 (Fisher’s exact)
Stenotrophomonas maltophillia	4	3	1	*p* = 0.317 (Fisher’s exact)
Chromobacter sakazzii	1	0	1	*p* = 1.000 (Fisher’s exact)

**Table 3:** Univariate analysis for the occurrence of infection on day 7 after admission.

	OR	95%CI	*p*-Value
Age	0.99	0.94–1.05	0.749
Gender			
Male	Ref.		
Female	0.98	0.28–3.51	0.980
MELD	1.08	0.97–1.21	0.159
APACHE II	0.96	0.85–1.08	0.504
Causes of cirrhosis			
Alcoholic cirrhosis	Ref.		
HBV + HCC	-	-	-
HBV + HDV	0.80	0.07–8.91	0.856
Autoimmune hepatitis	3.00	0.47–19.17	0.246
PCT	1.05	0.91–1.22	0.488
WBC	0.99	0.99–1.00	0.293

Footnotes: OR: odds ratio, CI: confidence interval, MELD: Model for End-Stage Liver Disease, APACHE II: Acute Physiology and Chronic Health Evaluation II, HBV: hepatitis B virus, HDV: hepatitis D virus, HCC: hepatocellular carcinoma, PCT: procalcitonin, WBC: white blood cell count.

**Table 4:** Day of infection onset.

	Total (*n* = 79) *n* (%)	Group A (*n* = 35) *n* (%)	Group Β (*n* = 44) *n* (%)	*p*-Value	
Occurrence of infection on day 7 after admission	10 (12.7)	2 (5.7)	8 (18.2)	0.172	Fisher’s exact test
Occurrence of infection on day 14 after admission	5 (6.3)	3 (8.6)	2 (4.5)	0.650	Fisher’s exact test
Occurrence of infection on day 21 after admission	5 (6.3)	1 (2.9)	4 (9.1)	0.376	Fisher’s exact test
Occurrence of infection on day 28 after admission	4 (5.1)	2 (5.7)	2 (4.5)	1.000	Fisher’s exact test

**Table 5:** Overall mortality and mortality in the two groups.

Total Mortality	Group A 9/35 (25.7%)	Group B 12/44 (27.3%)	*p* = 0.086 (Chi-Square, χ^2^ = 0.024)
Total mortality from infection in both Groups	6/79 (7.6%)	*p* = 0.039 (McNemar test)	
Total mortality from non-infectious diseases in both Groups	15/79 (19%)		
Total mortality from infection in Group A 1/35 (2.9%)	Total mortality from non-infectious diseases in Group A 8/35 (22.8%)		*p* = 0.039 (McNemar test)
Total mortality from infection in Group B 5/44 (11.4%)	Total mortality from infection in Group B 7/44 (15.9%)		*p* = 0.774 (McNemar test)

## Data Availability

The datasets used and/or analyzed during the current study are available from the corresponding author on reasonable request.

## Abbreviations

The following abbreviations are used in this manuscript:

MDR	Multidrug-resistant
XDR	Extensively drug-resistant
SSIs	Surgical site infections
ICU	Intensive care unit
AUTH	Aristotle University of Thessaloniki
CRP	C-reactive protein
PCT	Procalcitonin
MIC	Minimum inhibitory concentration
ESBL	Extended spectrum beta-lactamase
CRE	Carbapenem-resistant Enterobacteriaceae
